# Algal cultivation in urban wastewater: an efficient way to reduce pharmaceutical pollutants

**DOI:** 10.1007/s10811-016-0950-0

**Published:** 2016-09-03

**Authors:** Francesco G. Gentili, Jerker Fick

**Affiliations:** 1 Department of Wildlife, Fish and Environmental Studies, Swedish University of Agricultural Sciences, 901 83 Umeå, Sweden; 2 Department of Chemistry, Umeå University, 901 87 Umeå, Sweden

**Keywords:** Algae, Nitrogen, Pharmaceuticals, Phosphorus, Wastewater

## Abstract

The purpose of this study was to investigate whether pharmaceutical pollutants in urban wastewater can be reduced during algal cultivation. A mixed population of wild freshwater green algal species was grown on urban wastewater influent in a 650 L photobioreactor under natural light and with the addition of flue gases. Removal efficiencies were very high (>90 %), moderate (50–90 %), low (10–50 %), and very low or non-quantifiable (<10 %) for 9, 14, 11, and 18 pharmaceuticals, respectively, over a 7-day period. High reduction was found in the following pharmaceuticals: the beta-blockers atenolol, bispropol, and metoprolol; the antibiotic clarithromycine; the antidepressant bupropion; the muscle relaxant atracurium; hypertension drugs diltiazem and terbutaline used to relive the symptoms of asthma. Regression analysis did not detect any relationship between the reduction in pharmaceutical contents and light intensity reaching the water surface of the algal culture. However, the reduction was positively correlated with light intensity inside the culture and stronger when data collected during the night were excluded. Algae cultivation can remove partially or totally pharmaceutical pollutants from urban wastewater, and this opens up new possibilities for treating urban wastewater.

**Electronic supplementary material:**

The online version of this article (doi:10.1007/s10811-016-0950-0) contains supplementary material, which is available to authorized users.

## Introduction

Environmental pollution due to excessive releases of nutrients and other chemicals in urban wastewater is increasingly recognized as a major threat to aquatic ecosystems globally. A strategy that could counter this threat is to use algal ponds or bioreactors. This approach is not new (Oswald and Gotaas 1957). However, it has recently attracted the interest of many scientists around the world, mainly due to the ability of algae to take up nutrients and remove pollutants from wastewater efficiently (Hoffman 1998; Sturm and Lamer 2011), and the possibility of producing high-energy biomass from them (Rawat et al. 2011; Park et al. 2011a). For example, García et al. (2006) found that total nitrogen and phosphorus contents in municipal wastewater can be reduced by 73 and 43 %, respectively, by using mini high-rate algal ponds in Spain, and in other cases, reductions of 90–95 % have been reported (Hoffman 1998; Ruiz-Marin et al. 2010). The potential of using microalgae to remove nitrogen and phosphorous from sewage during tertiary treatment has also been assessed extensively (Pittman et al. 2011). Furthermore, in hectare-scale trials, high-rate algae ponds fed with primary settled wastewater have removed ca. 65 % of ammoniacal nitrogen and ca. 19 % of dissolved reactive phosphorus (Craggs et al. 2012). Algae have also been grown on other types of wastewater such as fish and animal production waste streams (Woertz et al. 2009; Riaño et al. 2011).

Pollutants that have received much attention recently include pharmaceuticals. These compounds are potent, biologically active chemicals, but little is known about their ecological effects, in contrast to the wealth of knowledge about their pharmacological and toxicological effects at high concentrations (Santos et al. 2010; Boxall et al. 2012). However, several studies have shown that pharmaceuticals enter waterways, primarily via treated wastewater effluent, and remain biochemically active in aquatic systems (Verlicchi et al. 2012; Hughes et al. 2013; Loos et al. 2013). They can also affect aquatic wildlife at environmentally relevant concentrations (Kidd et al. 2007; Brodin et al. 2013). Moreover, although municipal sewage water is commonly treated with a combination of mechanical, biological, and chemical processes before further release into the aquatic environment, this is not usually sufficient to eliminate pharmaceutical residues (Radjenović et al. 2009; Gros et al. 2010; Verlicchi et al. 2012). Thus, a number of additional promising treatments, such as ozonation, filtration through activated carbon, UV irradiation, H_2_O_2_ dosing, and/or retention in constructed free-water surface wetlands, have been suggested for improving removal efficiency (Joss et al. 2008; Matamoros et al. 2008; Breitholtz et al. 2012). It has also been shown that levels of veterinary antibiotics can be reduced in high-rate algal ponds fed with synthetic wastewater (de Godos et al. 2012). Recent studies have shown that *Chlorella sorokiniana* can greatly reduce paracetamol and salicylic acid added to an artificial medium under laboratory conditions (Escapa et al. 2015) and several other micropollutants from urine and anaerobically digested black water (de Wilt et al. 2016). Moreover, in a study involving growing algae on municipal wastewater in mini high-rate algal ponds during cold and warm seasons in Spain, the ability of algae to remove emerging organic contaminants was demonstrated (Matamoros et al. 2015). Biodegradation and photolysis have been suggested as the main removal pathways for micropollutants and emerging organic contaminants including pharmaceuticals (Matamoros et al. 2015; de Wilt et al. 2016). Interestingly, in another study, it was shown that biosorption, represented by the physico-chemical adsorption that occurs at the cell surface, was an important removal pathway of a biocide in both dead and living algal cells (Tam et al. 2002).

The continuing increases in atmospheric CO_2_ levels and associated demand for environmentally friendly sources of energy have drawn attention to another potential application of algae: their cultivation using the CO_2_ in flue gases to directly reduce emissions on site (Hughes and Benemann 1997; Chiu et al. 2011). From an environmental perspective, algae are particularly interesting since they can be used simultaneously for wastewater treatment, CO_2_ abatement (Wang et al. 2008), and bioenergy production (Craggs et al. 2012). However, few studies have considered the potential utility of cultivating algae to simultaneously treat flue gases and municipal wastewater (Kumar et al. 2010).

The aims of this study were to cultivate a mixed algal population for a week and evaluate the removal efficiency of selected pharmaceuticals.

## Materials and methods

### Experimental setup

A mixed population of wild freshwater green algal species has been growing for 3 years, following initial inoculation with *Tetradesmus dimorphus*, in an open photobioreactor (surface area 2.72 m^2^, volume 650 L) in a greenhouse on the roof of the combined heat and power plant in Umeå, northern Sweden (63°52′ N) (Axelsson and Gentili 2014). The reason for growing algae for 3 years was to follow the nutrient removal and algal population composition over a long period of time (unpublished data). The photobioreactor was constructed following the open-pond principle, where water flow is generated by a mechanical device. It was supported by a metal frame and constructed of thin fiberglass to allow light penetration not only from the top (as in a traditional open pond) but also from the sides and the bottom. The photobioreactor had the shape of an open pond with the following dimensions: 3 m long, 1.45 m wide, and 0.4 m deep with rounded corners (the water level was kept at 0.3 m); it had an empty space in the middle 2.1 m long and 0.6 m wide. The empty space was left to allow better illumination of the photobioreactor. Municipal wastewater influent was collected from the local wastewater treatment plant (Umeva, Umeå) and transported once a week to the power plant station in a 1-m^3^ tank, which was also used for partial settling of the influent to remove the large heavy particles before it was introduced into the photobioreactor.

Flue gases from the combined heat and power plant (Umeå Energi, Umeå), which burns both municipal and industrial solid wastes, were pumped from the smokestack and bubbled through the algal culture via a ceramic tubular gas diffuser (Cole-Parmer, USA) at a flow rate of approximately 3 L min^−1^. The CO_2_ concentration of the flue gases was measured every hour throughout the study period and had a mean value of 9 % ±1.8 SD (data received from Umeå Energi). The pH in all batches was 8.3 ± 0.9. Bubbling was stopped at night because without photosynthesis the algae culture would have been acidified. To study the performance of the algae under different environmental conditions such as natural light and temperature, the experiments were run from the beginning of April to the beginning of May 2012. At the start of every batch, 520 L of the previous batch was discarded and replaced by the same amount of new influent. At the start of five batches, and after 7 days, a 50-mL sample was collected from the photobioreactor. The collected sample was divided into two aliquots; one of 5 mL for biomass determination (see below) and the other of 45 mL that was centrifuged at 3580×*g* for 10 min; then, the supernatant was transferred to a clean tube for chemical analysis (see below), and this and the original tube containing the pelleted biomass were stored at −20 °C. In addition, the algal population in several samples was examined under a light microscope to identify species present as previously described (Gentili 2014). Influents that were not exposed to algae, but otherwise treated identically, were used as controls.

We decided to cultivate the mixed algal population for a week-long batch based on several previous experiments on a smaller scale with different algal strains performed for 6–8 days with excellent nutrient reduction (unpublished data). Furthermore, constructed free-water surface wetlands (CFWSWs) offer a relevant comparison with our system, since the retention times of approximately 1 week are similar (Breitholtz et al. 2012).

### Chemical analysis

Inorganic nitrogen contents in the samples were measured using ion-selective electrodes for ammonium (Cole-Parmer, USA) and nitrate (perfectION, Mettler Toledo, Switzerland). These analyses were performed with all five batches resulting in five replicates. During the nitrate measurements, an interference-suppressing solution was added to the samples following the electrode manufacturer’s instructions. Total phosphorus contents in three batches (three replicates) were measured using a phosphorus photometer and reagent kit (Hanna, Italy), following the manufacturer’s recommended protocol.

To detect and measure pharmaceuticals, 10- mL aliquots of the samples were filtered, using a 0.2-μm sterile filter (Sarstedt, Germany). Internal and surrogate standards (listed below) were then added, and the preparations were acidified to a pH of 3 using formic acid. Their pharmaceutical contents were then analyzed by liquid chromatography-tandem mass spectrometry (LC-MS/MS), as described in Lindberg et al. (2014). The LC-MS/MS system consisted of a PAL HTC autosampler (CTC Analytics AG, Switzerland), two pumps (Surveyor and Acella), and a mass analyzer (TSQ Quantum Ultra EMR, triple-stage quadrupole MS/MS, Thermo Fisher Scientific, USA), operating in positive or negative electrospray ionization mode. Concentrations of 79 pharmaceuticals were detected. Specific details related to the determination of these pharmaceuticals, including their ionization potentials, polarities, precursor/product ions, collision energies, tube lens values, quantification/qualification ions, and relative abundances, can be found in Grabic et al. (2012) and Lindberg et al. (2014). The system was based on column switching, using 6- and 10-port valves, with an injection volume of 1.0 mL using a 1- mL loop. In each analysis, 1.0 mL of acidified sample was injected, and the analytes were extracted using an OASIS HLB (20 mm × 2.1 mm i.d., 15 μm particle size) column, then separated using a fully endcapped C18 Hypersil GOLD aQ (50 mm × 2.1 mm i.d., 5 μm particles, Thermo Fisher Scientific, USA) column, following a corresponding guard column (20 mm × 2.1 mm i.d, 5 μm particles). Formic acid (Sigma-Aldrich, Germany) was used (at 0.1 %) to prepare the mobile chromatographic phases.

### Chemicals

All of the reference pharmaceutical standards were classified as analytical grade (>98 %). ^2^H_6_-amitriptyline, ^2^H_10_-carbamazepine, ^13^C_3_^15^N-ciprofloxacin, ^13^C_2_-ethinyl estradiol, ^2^H_5_-fluoxetine, ^13^C_6_-sulfamethoxazole, ^13^C^2^H_3_-tramadol, and ^13^C_3_-trimethoprim were obtained from Cambridge Isotope Laboratories (USA). ^2^H_5_-oxazepam, ^2^H_4_-risperidone, and ^13^C_2_^15^N-tamoxifen were purchased from Sigma-Aldrich (Germany). ^2^H_6_-codeine, ^2^H_4_-diclofenac, ^2^H_4_-flecainide, ^2^H_3_-ketoprofen, ^13^C_3_^2^H_3_-naproxen, and ^2^H_3_-paracetamol were purchased from CDN-Isotopes (Canada).

LC/MS grade methanol and acetonitrile (LiChrosolv–hypergrade) were purchased from Merck (Germany). Purified water was prepared using a Milli-Q Advantage system, including an ultraviolet radiation source (Millipore, USA).

### Instrumental analysis and biomass determination

Photosynthetically active radiation (PAR) was measured and recorded every 5 min using a LiCor 1400 datalogger connected to two spherical (6.1 cm diameter) LI 193 light sensors (LiCor, USA). One sensor measured the external light intensity in the air just above the photobioreactor. The other was placed in the algal culture, with its top 6 cm below the surface, to measure penetrating light. Samples of algal biomass were harvested by centrifuging 5- mL aliquots of the culture (3580×*g*, 10 min). The supernatant was then discarded, and the biomass was transferred to preweighed aluminium cups, dried at 70 °C for 24 h, and then weighed. Biomass was expressed as mean ± SE of TSS (g L^−1^) of five replicates (batches).

The dissolved oxygen content and temperature of the cultures were measured and recorded every 5 min using a ProODO optical dissolved oxygen sensor (YSI, USA).

### Statistical analysis

Removal efficiencies for nitrogen, phosphorus, and the detected pharmaceuticals were calculated from the difference in their concentrations in the influent before and after a 7-day exposure in the photobioreactor. Levels below the LOQ were replaced with corresponding LOQ values. The differences were analyzed using one-way analysis of variance and regression analysis, setting a 95 % confidence level (Minitab 16.1.0).

## Results

### Algal species

Following numerous changes of wastewater during the 3 years since its initial inoculation with *Tetradesmus dimorphus*, the algal population in the bioreactor on the roof of the power plant has changed substantially. The most frequent genus in the batches we examined was the green alga *Dictyosphaerium*.

### Influent characteristics, nutrient removal, and biomass production

The municipal influent had mean (±SE) inorganic nitrogen, total phosphorus, and total suspended solid (TSS) contents of 49.7 ± 12.2 mg L^−1^, 2.4 ± 0.9 mg L^−1^, and 0.076 ± 0.022 g L^−1^, respectively. At the end of the batches, inorganic nitrogen contents were reduced on average by 67.8 ± 2.7 %. Total phosphorus contents were reduced on average by 55.6 ± 10 %. Biomass production at the end of the batches had a mean (±SE) value of 0.22 ± 0.03 g L^−1^ TSS.

The CO_2_ bubbling has had two important functions such as the addition of carbon as well as the regulation of the pH at a value of 8.3 ± 0.9 through the entire experiment.

### Abiotic factors

**Fig. 1:**
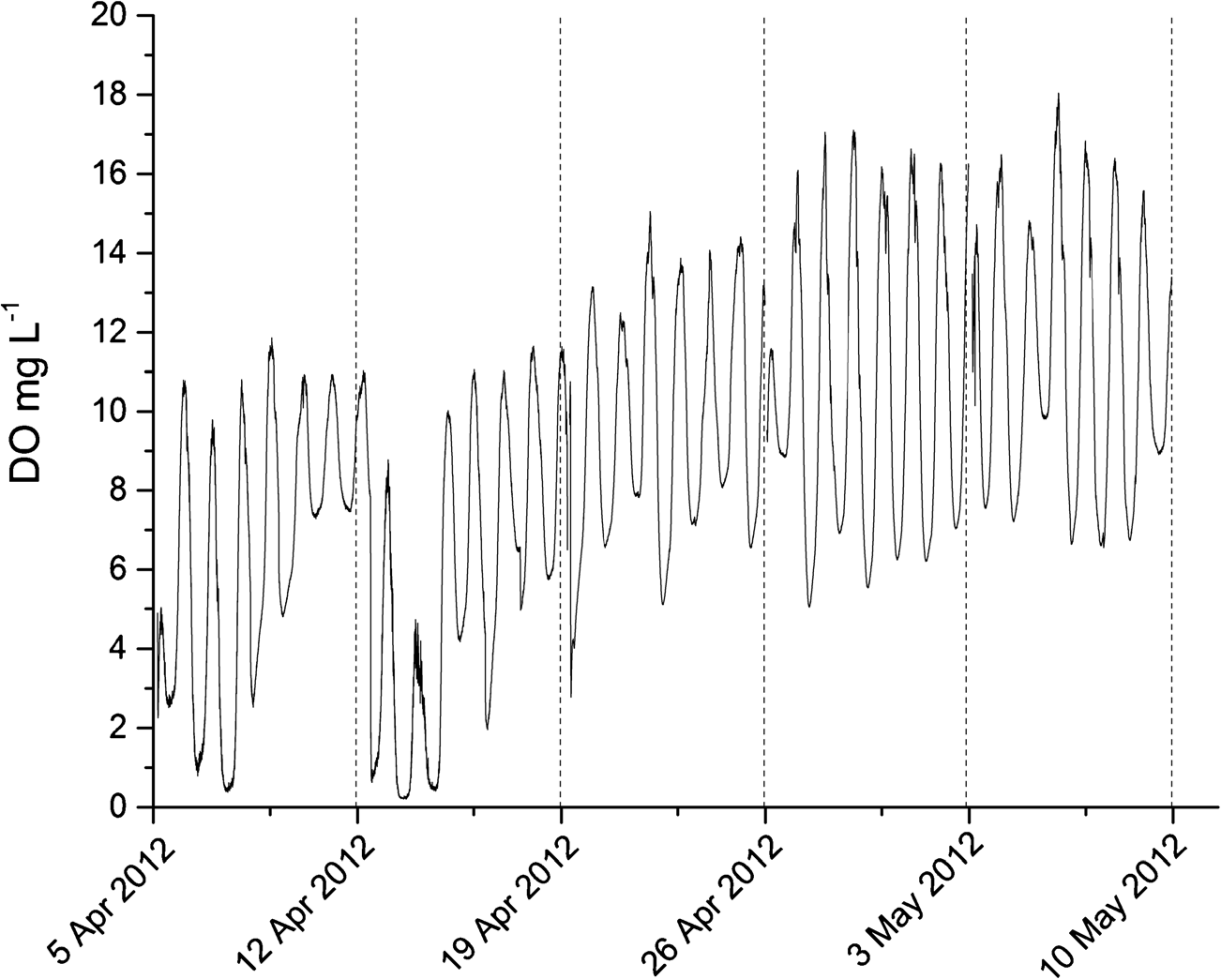
Dissolved oxygen (DO) measured every 5 min throughout the experiment

Inside the algal culture, PAR varied (Table 1) due to changes in the light intensity in the air, the concentration, and the clustering of algal biomass. Temperature varied greatly throughout all the experiments (all batches), ranging between 10 and 32 °C with a mean of 18 °C. Dissolved oxygen contents ranged from 0.2 to 18 mg L^−1^, increasing with time during both individual batches and the entire experiment (Fig. 1). The same trend was observed when dissolved oxygen data were normalized with respect to temperature (data not shown). Dissolved oxygen was significantly correlated to nitrogen removal (Table 3).

**Table 1:** Light inside and outside the algae culture was measured every 5 min throughout the experiment

Batch	Light outside photons μmol m^−2^ s^−1^	Light inside photons μmol m^−2^ s^−1^
1: April 5–12	352.7	15.5
2: April 12–19	462.9	16
3: April 19–26	293.5	20.8
4: April 26–May 3	500.4	25.8
5: May 3–10	465.2	27.7

The values represent the mean for each batch

### Reductions in pharmaceutical contents

The analytical method we used was stable throughout the study: all retention times were within 2 % of the standards, and we detected no memory effects or cross-talk. Of the 79 pharmaceuticals included in the study, 27 were not detected in any sample (names, quantification limits, and measured levels of all pharmaceuticals in all samples are presented in the supplementary data, Tables S1 and S2). Levels ranged from 0.16 to 2.9 μg L^−1^ with a mean of 217 ng L^−1^ and a median of 59 ng L^−1^. Removal efficiencies, measured as the difference between untreated influent before and after a 7-day exposure in the photobioreactor, for 52 pharmaceuticals are presented in Table 2. Removal efficiencies were very high (>90 %), moderate (50–90 %), low (10–50 %), and very low or non-quantifiable (<10 %) for 9, 14, 11, and 18 pharmaceuticals, respectively (Table 2).

**Table 2:** Removal efficiency of the pharmaceuticals expressed as mean ± standard deviation of the five batches with algae and control without algae

	With algae 7 days			Without algae day 7
	Mean (%)	SD	n=	
Alfuzosin	64	*28*	*5*	−2.3
Alprazolam	−49	*210*	*3*	12
Atenolol	99	*0.61*	*5*	14
Atracurium	97		*2*	<LOQ
Azelastine	27		*1*	<LOQ
Biperiden	−490	*1000*	*4*	66
Bisoprolol	97	*2,6*	*4*	−2.8
Bupropion	93	*12*	*4*	36
Carbamazepin	−14	*6.1*	*5*	−53
Cilazapril	61		*1*	<LOQ
Ciprofloxacin	11	*46*	*5*	−5.6
Citalopram	98	*0.37*	*5*	−33
Clarithromycine	90	*13*	*5*	−73
Clemastine	40		*1*	<LOQ
Clindamycine	45	*25*	*5*	−8.8
Clonazepam	88		*2*	−473
Clotrimazol	19		*1*	<LOQ
Codeine	−11	*61*	*4*	22
Cyproheptadine	−450		*1*	8.7
Desloratidin	−45	*270*	*4*	−1047
Dicycloverin	71		*2*	2.5
Diltiazem	94	*4.2*	*5*	11
Diphenhydramin	89	*15*	*3*	15
Eprosartan	80	*21*	*4*	82
Fexofenadine	−5.2	*48*	*5*	−81
Flecainide	58	*14*	*5*	−111
Fluconazole	−17	*24*	*5*	28
Flupetixol	−75		*2*	<LOQ
Haloperidol	−5000	*4400*	*3*	−21
Hydroxyzine	87	*8.2*	*4*	<LOQ
Ibersartan	6.4	*130*	*5*	−7.6
Loperamide	41	*29*	*3*	<LOQ
Memantin	81	*13*	*5*	3,6
Metoprolol	99	*1,8*	*5*	19
Miconazole	65		*2*	48
Mirtazapine	39	*22*	*5*	−8.8
Nefazodon	−630		*1*	−15
Orphenadrin	−3.8	*75*	*5*	93
Oxazepam	−13	*34*	*5*	−6.3
Pizotifen	80		*2*	<LOQ
Ranitidine	75	*28*	*3*	24
Risperidone	−3.2	*6.4*	*3*	−169
Roxithromycine	44		*2*	<LOQ
Sertraline	17	*14*	*3*	<LOQ
Sotalol	43	*37*	*5*	59
Sulfamethoxazol	6.0	*32*	*5*	25
Terbutalin	98		*1*	11
Tramadol	57	*33*	*5*	17
Trihexyphenidyl	49	*67*	*4*	<LOQ
Trimetoprim	3.7	*11*	*5*	−45
Venlavafaxin	57	*32*	*5*	40
Verapamil	−13		*1*	<LOQ

*<LOQ* below limit of quantification

Initial concentrations for each pharmaceutical in each batch are given in supplementary Table S1.

Several pharmaceuticals were degraded more efficiently in the algal photobioreactor than in the influent treated without algae. On average, only 8 pharmaceuticals were not removed or had apparently negative removal rates in the presence of algae, compared to 20 without algae. Furthermore, the average removal efficiency for those that were removed increased from 30.3 to 61.3 % in the presence of algae (Table 2). For example, beta-blockers were not degraded at all without algae and, with the exception of sotalol, were almost completely removed in the algal bioreactor.

Regression analysis did not detect any relationship between the reduction in pharmaceutical contents and light intensity just outside the photobioreactor (and thus reaching the water surface of the algal culture; Table 3). However, the reduction was positively correlated with light intensity inside the culture and was stronger when data collected during the night were excluded. Dissolved oxygen contents and the reduction in nitrogen contents were also positively correlated with the reduction in pharmaceutical contents.

**Table 3:** Results from the regression analysis and analysis of variance between the reduction of pharmaceutical contents (Pharm) in the five batches and explanatory factors, including: “outside light” and “inside light” (the mean photosynthetically active radiation, PAR, during the experiment recorded by the sensors located just above the photobioreactor and immersed in the algal culture, respectively); “direct outside light” and “direct inside light” (mean PAR during the time with direct sunlight); “N red” (the reduction in nitrogen content); and “DO” (dissolved oxygen content)

Interaction	*R* ^2^	*p* value
Pharm × outside light	0.0	0.422
Pharm × direct outside light	0.0	0.879
Pharm × inside light	0.82	0.022
Pharm × direct inside light	0.92	0.006
Pharm × temperature	0.0	0.406
Pharm × DO	0.74	0.038
Pharm × N red	0.82	0.021
N red × DO	0.78	0.03

## Discussion

Interestingly, *Dictyosphaerium*, the most frequent alga found in the present study, was among the dominant algae species in a wastewater treatment study performed in New Zealand (Park et al. 2011b). This could complicate harvesting by sedimentation (rates of which are strongly influenced by the size of cells and colonies), since *Dictyosphaerium* can remain suspended for a long time (Park et al. 2011b).

The inorganic nitrogen and total phosphorus removal were similar to those previously recorded in mini high-rate algal ponds fed with municipal wastewater in Spain (García et al. 2006). A higher ammoniacal nitrogen removal than dissolved reactive phosphorus was previously found in high-rate algal ponds (Craggs et al. 2012). Furthermore, the present study reported the removal of total phosphorus, which included easily available phosphate but also less available phosphorus fractions. In another study, the green microalga *Chlorella sorokiniana* could completely remove nitrogen and phosphorus from anaerobically digested black water (de Wilt et al. 2016); however, the alga was grown in flasks under laboratory conditions with continuous light for 31 days.

The levels of total suspended solids were lower in our influent samples than in typical municipal wastewater influent due to settling in the tank used for transportation, but the levels at harvest time (0.22 g L^−1^) were comparable to levels found in a similar study (García et al. 2006). However, there are several differences between the studies, such as location, and the use of flue gases in the present study but not in the cited study. Furthermore, the dominant algae species in the present study was *Dictyosphaerium*, but the algae species present in the cultures analyzed by García et al. (2006) were not reported.

There were large diurnal variations in dissolved oxygen contents due to diurnal variations in solar radiation, as reported in another study (García et al. 2006). However, in our study, the diurnal variations in dissolved oxygen contents decreased over time during the first three batches. This was presumably because the algae were least dense and the organic matter content highest at the beginning of each batch, and thus, relatively, little oxygen was evolved and high quantities were consumed in the breakdown of organic matter. Nevertheless, even the minimum oxygen levels were detectable, thus oxygen partial pressures should not limit degradation of organic matter in our system.

The positive correlation between DO and pharmaceutical removal confirms what was previously found where the reduction of the beta-blocker atenolol was higher under an aerobic regime than under a microaerobic regime (Stadler et al. 2015).

In our study, the temperature did not have any clear effect on pharmaceutical removal (Table 3). In another study performed in Spain, Matamoros et al. (2015) showed that pharmaceutical removal efficiency was higher during the warm season than during the cold season; however, the difference from warm and cold seasons was not only represented by temperature but by light intensity as well.

Some pharmaceuticals were detected at higher levels in the outgoing water than in the incoming water, and some were only detected in outgoing water and not at all in incoming water in this study, resulting in negative removal rates (Table 2). This could partly be due to analytical variations, since most of these observations occurred close to the LOQs. Many pharmaceuticals are also metabolized and excreted as glucuronides or other conjugated metabolites, and these can be converted to the parent compound by enzymatic processes. Deconjugation has been shown to occur in sewage treatment processes, and negative removal efficiencies have been shown for macrolide antibiotics, carbamazepine, and other pharmaceuticals (e.g., Vieno et al. 2007; Gros et al. 2010).

In a previous study, the beta-blocker metoprolol was degraded from 60 to 100 % after 23–31 days of *C. sorokiniana* cultivation under laboratory conditions, and it was suggested that the degradation was due to biodegradation and photolysis (de Wilt et al. 2016). In another study, the reduction of two pharmaceuticals (paracetamol and salicylic acid) by the green alga *C. sorokiniana* was shown (Escapa et al. 2015). However, Escapa et al. (2015) grew the algae under laboratory conditions with artificial light and on an artificial medium with the addition of two pharmaceuticals, while in the present work, we grew algae in a large volume of municipal influent with the addition of flue gases from the power plant and under natural illumination. Despite large differences, both studies show a great reduction of pharmaceuticals by microalgae growth.

Our results concerning pharmaceutical removal are in line with a study involving growing algae on municipal wastewater during the cold and warm season in Spain (Matamoros et al. 2015). It has been suggested that photolysis is one of the most important removal pathways for several micropollutants including pharmaceuticals (Matamoros et al. 2015; de Wilt et al. 2016). This removal action found confirmation in our results where pharmaceutical removal and light inside the algae culture were closely correlated (Table 3), although our control exposed to light without algae did not show such a reduction. Other important removal pathways are biodegradation (Matamoros et al. 2015; de Wilt et al. 2016) and biosorption (Tam et al. 2002). Interestingly, biosorption and biodegradation are algal species specific as shown for *Chlorella* and *Tetradesmus* (Tam et al. 2002). In our case, although the predominant algal species was *Dictyosphaerium*, other algal species were present. It is important to bear in mind that biodegradation and biosorption of micropollutants such as pharmaceuticals and biocides can be influenced not only by population density, but even more by the specific ability of the algal species to adsorb and/or absorb and degrade the pollutants. Hence, more studies are needed to characterize more species and mixed populations for their ability to adsorb, absorb, and degrade several micropollutants.

Another option for improving the removal efficiencies of pharmaceuticals and other pollutants is to use constructed free-water surface wetlands (CFWSWs). The removal rates of 92 pharmaceuticals in four CFWSWs recorded by Breitholtz et al. (2012) provide particularly relevant comparisons with rates we observed in our photobioreactor, since the retention times of approximately 1 week are similar, and both studies were conducted in early spring at similar latitudes. The removal rates of 34 pharmaceuticals were recorded in both studies, and for 11 of these, the removal rates were higher in our photobioreactor than in the CFWSWs (Breitholtz et al. 2012), and substantially higher for several pharmaceuticals. Notably, the removal rates were low or non-detectable in the studied CFWSWs (Breitholtz et al. 2012), but high in the algal bioreactor for: the beta-blockers atenolol, bispropol, and metoprolol; the antibiotic clarithromycine; the antidepressant bupropion; and the hypertension drugs diltiazem and memantine which are used to treat Alzheimer’s disease.

## Electronic supplementary material

Table S1 (XLS 56 kb)

Table S2 (XLS 18 kb)
